# Effects of calcium supplementation on changes in the IL2, IL4, IL6, IL10 axes and oxidative stress in pregnant women at risk for pre-eclampsia

**DOI:** 10.1186/s12884-023-06235-8

**Published:** 2024-01-20

**Authors:** Erica de Brito Pitilin, Filomena Marafon, Beatriz da Silva Rosa Bonadiman, Bruno Bordin Pelazza, Micheli Mainardi Pillat, Jéssica Dotto de Lara, Patrícia Pereira de Oliveira, Margarete Dulce Bagatini, Janine Schirmer

**Affiliations:** 1 Graduate Program in Nursing, Federal University of Fronteira Sul, Chapecó, Santa Catarina Brazil; 2 Graduate Program in Biomedical Sciences, Federal University of Fronteira Sul, Chapecó, Santa Catarina Brazil; 3 Graduate Program in Nursing, Midwest State University, Guarapuava, Paraná Brazil; 4 Graduate Program in Pharmaceutical Sciences, Department of Microbiology and Parasitology, Federal University of Santa Maria, Santa Maria, Rio Grande Do Sul Brazil; 5 Community University of Chapecó, Chapecó, Santa Catarina Brazil; 6 Graduate Program in São Paulo School of Nursings, Federal University of São Paulo, São Paulo, SP Brazil

**Keywords:** Endothelial dysfunction, Pregnancy, Calcium, Pre-eclampsia, Supplementation

## Abstract

**Background:**

Pregnant women with hypertensive disorders are at increased risk for inflammatory diseases and oxidative stress. The dilemma raised by the best dosage of calcium supplementation on these factors is evident. The aim of the current study was to examine the effects of calcium on biomarkers of the purinergic system, inflammation and oxidative stress, which are factors contributing to vascular damage in pregnant women at high risk of pre-eclampsia.

**Methods:**

A prospective, double-blind and placebo-controlled study conducted with 101 women at risk of pre-eclampsia were randomized to take 500 mg calcium/day or 1,500 mg calcium/day or placebo for 6 weeks from the 20th gestational week until delivery. Fasting blood samples were collected at the beginning of the study and 6 weeks after the intervention.

**Results:**

Taking calcium supplements (500 mg calcium/day) led to a significant increase in ATP hydrolysis (*p* < 0.05), NTPDase activity with increased hydrolysis of ADP and AMP nucleotides in platelets and lymphocytes. In the intragroup analysis IL-2, IL-6, IL-4 and interferon-ɣ presented lower values in the calcium 1,500 mg/day group (*p* < 0.005). Oxidative stress was assessed by TBARS pro-oxidant marker, with an increase for the calcium groups when compared to the placebo group. The Vitamin C antioxidant marker presented a significant increase (*p* < 0.005) for the group that received high calcium doses.

**Conclusions:**

Calcium administration for 6 weeks had antioxidant action and positively modulated the purinergic system and inflammatory markers in pregnant women at risk of pre-eclampsia.

**Supplementary Information:**

The online version contains supplementary material available at 10.1186/s12884-023-06235-8.

## Introduction

Normal fetal development depends on maternal hemodynamics and on cardiovascular changes to meet the increasing oxygen and nutrient demands [1]. Alterations in these adaptive changes can develop gestational hypertensive disorders that affect from 5 to 30% of the pregnancies, contributing to nearly 60,000 maternal deaths/year due to complications inherent to these disorders, constituting the leading cause of maternal mortality in low-income and still developing countries like Brazil [2].

Altered placental implantation has traditionally been accepted as the primary event responsible for the histological abnormalities seen in pre-eclampsia [3]. Ischemic placenta releases antiangiogenic factors into the mother's circulation, causing widespread endothelial damage, high levels of inflammatory cytokines and increased oxidative stress, factors that affect the endothelial function in the entire maternal circulation system with detrimental consequences for fetal health [4, 5].

Although etiology and pathogenesis of these diseases remain unclear, advances indicate that organ deterioration processes share common essential characteristics in pre-eclampsia, including cellular injury and increased plasma biomarkers such as 8-isoprostane, C-reactive protein, IL-1β, IL-6 and IL-10, in addition to other dysfunctions that contribute to functional development and morphological impairment of the cells [6, 7].

A recent study showed that adverse pregnancy outcomes such as fetal growth restriction (FGR), placenta previa, placental abruption and pre-eclampsia were associated with increased serum levels of soluble fms-like tyrosine kinases (sFlt-1), 8-epi-prostaglandinF2-alpha (8epi-PGF2α) and decreased levels of the placental growth factor (PlGF) and of total antioxidant capacity (TAC), in addition to vascular endothelial damage, cardiovascular complications and an exaggerated inflammatory response [8, 9].

Despite the considerable progress made in understanding the molecular mechanisms of these diseases, the current therapeutic options are limited and no pharmacological treatment has emerged to date. However, calcium supplementation suggests a promising beneficial effect in gestational hypertensive disorders, capable of modulating vascular metabolism and reducing the risk of mortality due to cardiovascular diseases by nearly 30% [10, 11].

Although the recommendation is to initiate calcium supplementation at the 20th gestational in at-risk pregnant women from low-income countries, universal calcium supplementation for pregnant women is not part of the prenatal care services in Brazil. Conflicting results have been reported on the safety of effective dosage, as well as of long-term and excess calcium supplementation in low- and middle-income countries [12, 13]. Therefore, the conflicting effects of the action of calcium fall on the recommended dosage and use time of the supplement.

To the present day, there is scarce evidence of biological mechanisms that link calcium supplementation to endothelial damage in hypertensive pregnant women [14]. Elucidation of common and unique mechanisms responsible for the deterioration present in hypertensive syndromes can facilitate identification and development of effective goals and therapies [6].

From the analysis of the purinergic system, it will be possible to understand such mechanisms by revealing possible markers or relevant pathways in these pathological processes and, in this way, this understanding can help us prevent complications by means of timely interventions, mainly those related to pre-eclampsia. Our starting point is the assumption that calcium supplementation contributes to increasing vasodilation and to mediating endothelial dysfunction in pre-eclampsia, as well as to reducing the inflammatory and oxidative stress processes.

This study aimed at evaluating the activity of purinergic ectoenzymes and oxidative stress and inflammation markers in hypertensive pregnant women supplemented with calcium at low and high doses.

## Methodology

### Participants

The study participants were pregnant women at high cardiovascular risk recruited from the reference outpatient service for high-risk prenatal care in the Brazilian South Region, linked to the Unified Health System (*Sistema Único de Saúde*, SUS). The territory chosen to develop the research is a reference in health actions and makes up the region of the Great Mercosur Border (Meso Mercosur).

### Experimental design

This is a placebo-controlled randomized clinical trial conducted with 3 parallel groups from June 2018 to July 2019. Randomization was performed by a statistician unrelated to the trial using an online calculator (http://randomization.com/) by means of permuted blocking. Treatment assignments within the blocks were determined randomly in order, with the desired allocation ratio achieved within each block: 1:1:1. The codes generated were allocated in packages containing the codings generated by randomization.

Sample size was calculated based on the primary results of calcium supplementation in pregnant women from previous studies^(1,4)^. The difference between the group means in the reduction of systolic and diastolic blood pressure and proteinuria (4% in the placebo group, 8% in the 500 mg calcium/day group, and 12% in the 1,500 mg calcium/day group) was considered, with Type I error (α) of 0.05%, Type II error (beta) of 0.20%, and 80% study power. The required sample calculated totaled 175 pregnant women. However, due to probable losses, sample calculation was expanded by 10%, totaling pregnant women, subdivided into three groups.

The inclusion criteria were as follows: pregnant women over 18 years of age, with a single fetus, primigravidas, diagnosed with gestational hypertension, overweight/obesity, at least 20th gestational weeks, low socioeconomic factor, with low dietary calcium intake (less than 800 mg/day), and who were not in use of medications that could interfere with calcium absorption (for example: corticosteroids, thiazides and thyroid hormones).

The diagnosis of pre eclâmpsia was characterized by gestational hypertension with increased blood pressure levels at or above 140 mmHg for systolic blood pressure (SBP) and at or above 90 mmHg for diastolic blood pressure (DBP) identified in Korotkoff phase V after the 20th gestational week, overweight/obesity, low socioeconomic factor and with low dietary calcium intake (less than 800 mg/day). The clinical evaluation was carried out in accordance with international recommendations and guidelines for the care for pregnant women at risk [10].

**Fig. 1:**
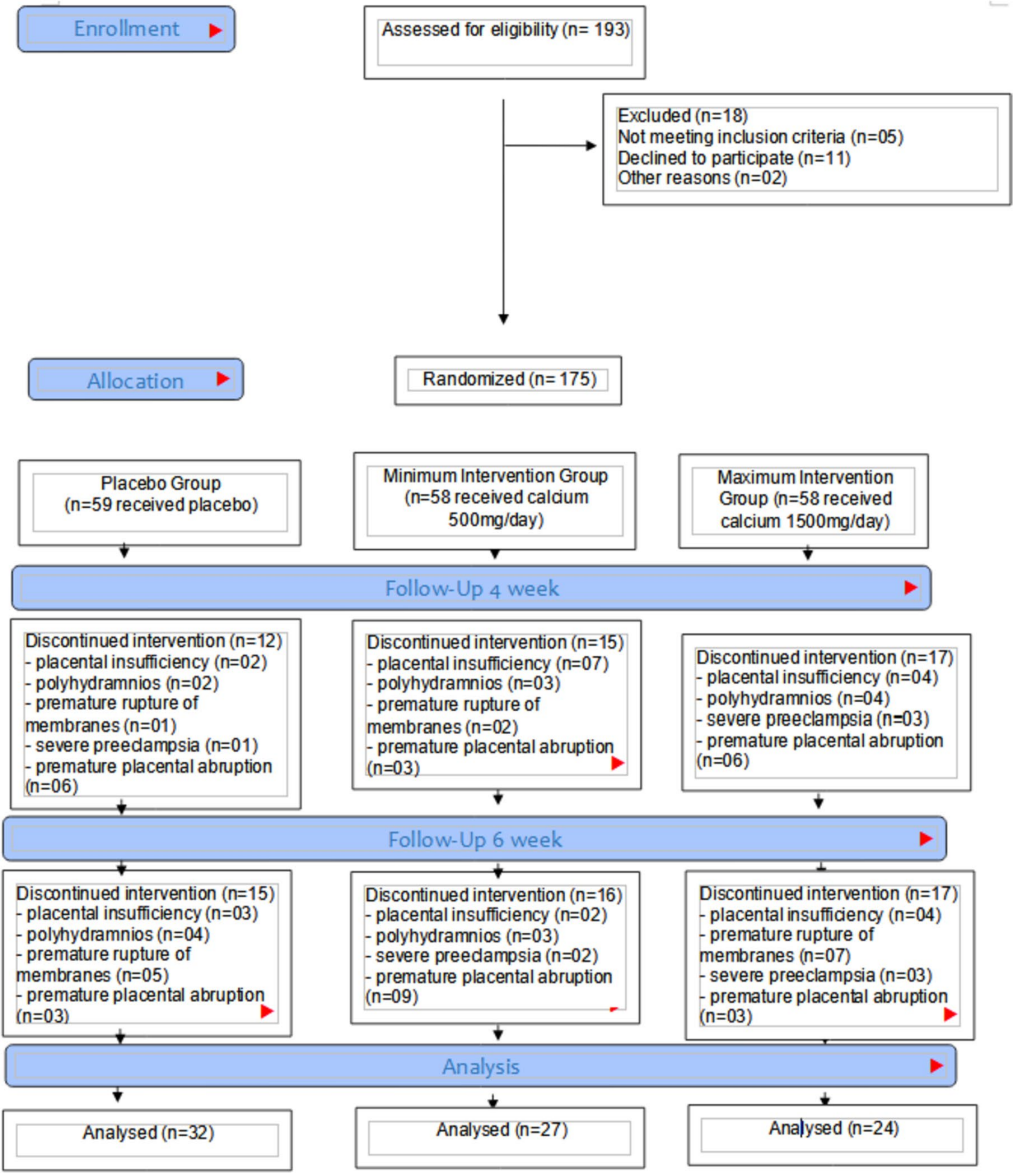
Flow diagram of the study design and participant allocation

After randomization, 92 participants were excluded from the study because they presented polyhydramnios, severe anemia, fetal death, history of hypertension before pregnancy, premature placental abruption, premature rupture of membranes (PROM) and altered umbilical artery Doppler, uteroplacental insufficiency (Fig. 1). These participants were at high risk of eclampia requiring hospitalization and the use of other medications that could interfere with calcium and therefore did not continue in the study.

### Randon

The participants were randomly allocated and masked regarding the dosage received from the 20th gestational week to delivery. A third person was responsible for administering and prescribing the tablets. The study researchers were not masked for calcium dosage because masking does not interfere with the primary outcome of this study, which consists in clinical and laboratory measurements.

### Intervention

The intervention consisted in prescribing 02 elemental calcium tablets containing 500 mg a day (500 mg calcium/day group), 02 elemental calcium tablets containing 1,500 mg a day (1,500 mg calcium/day group) and 02 placebo tablets for the control group. The placebo tablet was indistinguishable from the calcium one in aspect, taste and dosage prescribed.

The tablets were provided to the participants in each follow-up instance of the study. It was instructed that calcium be consumed with water, preferably between both main meals (lunch and dinner), in order to minimize any possible interference with the absorption of other minerals. No participant was administered or made use of anti-hypertensive medications during the study period.

The pregnant women were instructed to continue supplementation until delivery, and were asked not to alter their normal dietary intake during the study and not to take supplements other than those provided to them by the researcher. Envelopes containing the tablets were handed in to the participants, with the exact number of tablets for 06 weeks.

### Ethics approval

The study complied with the required ethical requirements, respecting the regulatory guidelines and norms for research with human beings. It is approved in the Brazilian Registry of Clinical Trials (https://ensaiosclinicos.gov.br/rg/RBR-9ngb95). Trial identification UTN code: U1111-1221–4052. Date of registration: 11/07/2018. Last approval date: 06/28/2022 Also, this study was perfomed in line with the principles of the Declaration of Helsinki. Approval was granted by the Ethics Committee of Federal University of São Paulo (17/05/2018 under n° 2.659.764). Informed consent was obtained from all individual participants included in the study. All experiments were performed in accordance with relevant guidelines and regulations.

All experimental protocols were approved by a named institutional and/or licensing committee by the Ethics Committee of Federal University of São Paulo. The consent was obtained from all subjects and/or their legal guardian(s).

### Outcomes

The primary outcome of the study was the evaluating the activity of purinergic ectoenzymes and oxidative stress and inflammation markers in hypertensive pregnant women supplemented with calcium at low and high doses.

Secondary outcomes were low birth weight (< 2.500 g) and prematurity (< 37 weeks).

### Reagents and equipment

The following were used to perform the protocols for analysis of the purinergic system: substrates ATP, ADP, AMP, Ado, bovine serum albumin, HEPES, Ficoll-Histopaque and Coomassie reagent obtained from Sigma-Aldrich (St. Louis, MO, United States of America – USA). The CBA (*Cytometric Bead Array Human Th1/Th2 Cytokine Kit II*) kit, obtained from BD Bioscienses (BD Diagnostics, Burlington, NC, USA), was used to quantify the interleukins. All the other reagents used in the experiments of the purinergic system and oxidative profile were obtained with analytical grade and high purity.

The centrifuge used is Sigma 3 k—16L®, the proteins were dosed and normalized in a UV/VIS 5300PC spectrophotometer (Shanghai Metash Instruments Co., Shanghai, China) and the other readings were made in a Multiskan™ GO microplate spectrophotometer (Thermo Fisher Scientific, Waltham, MA, USA). A BD Accuri C6 flow cytometer (BD Diagnostics, Burlington, NC, USA) was used for the analysis of the inflammatory parameters. All the samples obtained were stored at -80 °C in an Indrel IULT 335D-486 ultrafreezer.

### Separation of biological samples

The biological material was collected by means of anterocubital venipuncture in EDTA tubes, sodium citrate tubes and tubes without anticoagulant to obtain serum, stored in thermal boxes and sent to the UFFS Biochemistry laboratory for processing. The tubes without anticoagulant were centrifuged at 3,500 rpm for 15 min and the serum obtained was stored in a cryotube and stored in an ultrafreezer at -80 °C.

To obtain the platelet concentrate, the whole blood tube collected with sodium citrate as anticoagulant was centrifuged at 1,200 rpm for 10 min. Subsequently, the plasma obtained was again centrifuged at 5,000 rpm for 30 min and washed with a 3.5 mM HEPES buffer, pH 7.2, according to the protocol [15, 16]. The concentrate content obtained was resuspended in a 500 μl HEPES buffer and stored in an ultrafreezer at -80 °C.

For separation of the lymphocyte layer, the adapted methodology [17] was employed, using the Ficoll-Histopaque reagent, which promotes separation by a density gradient. For the separation procedure, the samples from the tubes containing EDTA were diluted in 0.9% saline solution and added to the tube containing Ficoll, which was centrifuged at 1,800 rpm for 30 min. After the centrifugation procedure, the layer gradient was observed, and the lymphocyte concentrate was removed from the intermediate layer and, subsequently, it was subjected to washing and centrifugation procedures with 0.9% saline solution. At the end, the supernatant was discarded and 500 μL of saline solution was added to the lymphocytes, storing the sample in the ultrafreezer to perform the analytical protocols.

### Protein dosage

The proteins of the samples were assessed by means of the Bradford method [18], with reading at 595 nm, using bovine serum albumina as standard. For protein dosage of the samples, 2,500 μL of the Coomassie reagent and 50 μL of sample were added in a test tube, absorbance was read and the concentrations were calculated.

The protein concentrations of the samples were normalized from 0.1 to 0.2 mg/mL and from 0.4 to 0.6 mg/mL, for lymphocyte and platelet samples, respectively. Values above the range defined were diluted with saline solution for the lymphocytes and with a HEPES buffer for the platelets.

### NTPDase and ecto-5'-nucleotidase activity in platelets and lymphocytes

The ATP hydrolysis into ADP and AMP test was performed to measure the activity of the NTPDase and ecto-5'-nucleotidase ectoenzymes, according to the methodology defined by Pilla [15] and adapted by [16], which evaluates the released inorganic phosphate reading in a spectrophotometer at 630 nm; the results were expressed as nmolPi/min/mg protein.

The test consists in incubating the sample and nucleotide (10 mM ATP/ADP and 20 mM AMP) with an incubation system containing 1.2 M sodium chloride, 60 mM glucose, 50 mM potassium chloride, 500 mM tris-hydrochloride (pH 7.4), 50 mM calcium chloride (for ATP and ADP), and 100 mM magnesium chloride (for AMP), followed by coloring with malachite green. The lymphocyte and platelet samples were incubated for 60 and 70 min, respectively.

### Activity of ADA in platelets and lymphocytes

Activity of the adenosine deaminase (ADA) ectoenzyme was determined according to the protocol defined by Giusti and Galanti [19], with readings of the amount of ammonia released performed in a spectrophotometer at 620 nm. Briefly, the protocol consists in making the sample react with 21 mM adenosine after incubation (at 37 °C for 60 min) and adding phenol/nitroprusside and alkaline hypochlorite solutions (at 37 °C for 30 min); the results were expressed in units per liter (U/L).

### Quantification of the interleukins

For determination of the inflammatory parameters, interleukins (IL) IL-2, IL-4, IL-6, IL-10, tumor necrosis factor (TNF) and interferon gamma (INF-ɣ) were quantified using a commercial *Cytometric Bead Array Human Th1/Th2 Cytokine Kit II* (CBA II), catalog code 551,809 from BD Bioscienses, according to the manufacturer's instructions. For the CBA II test, the samples were labeled with a set of analytes of known size and fluorescence, assembling sandwich complexes that were sequentially captured in a flow cytometer; the results were expressed in pg/mL.

### Oxidative stress markers

The oxidative profile markers of the pregnant women were evaluated from the plasma samples with EDTA and corresponded to oxidative stress indicators such as thiobarbituric acid reactive substances (TBARS) and myeloperoxidase (MPO), and to markers of the enzymatic and non-enzymatic antioxidant defenses, namely: ascorbic acid (Vitamin C), protein (PSH) and nonprotein (NPSH) thiols.

For the TBARS test, a measurement was taken of the amount of malondialdehyde released by the lipoperoxidation process caused by the reactive oxygen species (ROS), which, when heated in the presence of thiobarbituric acid, forms a pink compound, which was measured in a spectrophotometer at 532 nm according to the protocol defined by Jentzsch [20] with modifications, expressing the results in nmol MDA/mL. The MPO test comprised quantification of this enzyme in a spectrophotometer at 492 nm, according to Suzuki [21] with modifications, and the results were expressed as mM quinoneimine produced in 30 min; MPO is produced by inflammatory mediators and released by the leukocytes at injury sites, generating hypochlorous acid.

Evaluation of the PSH and NPSH antioxidant markers was performed according to the Ellman [22] protocol with modifications, where the thiol groups react with reagent 2,2’-dinitro-5,5’-dithiodibenzoic acid (DTNB) generating a yellow compound, which is read in the spectrophotometer at 412 nm, expressing the result in μmol/mL of plasma. To assess the exogenous antioxidant, Vitamin C, the protocol defined by Jacques-Silva [23] with modifications, absorbance was read at 520 nm and the results were expressed in grams/liter (g/L).

### Data analysis

The analyses were performed using the *Statistical Package for the Social Sciences* (SPSS) software, version 20.0. Automatic data verification was performed at the typing moment, through the *Check* function. To identify and correct inconsistencies in coding, reviewing and typing, data debugging was performed, obtaining the frequencies of the variables collected in the program itself.

For the longitudinal analyses, repeated evaluations of each patient were used for all the outcomes (dependent variables) in generalized linear mixed models. Use of GLMM considers the totality of observations, even those corresponding to the discontinued patients. The model allowed assessing the time (before and after the intervention) and group (calcium 500 mg/day, calcium 1,500 mg/day and placebo) fixed factors, as well as a possible interaction effect between time and group.

The model was fitted with gamma distribution with log-link, considering individuals as random effect and first-order autoregressive covariance matrix (AR1). The best fit was defined by means of the Akaike Information Criterion (AIC). The Normal and Tweedle probability functions were also tested. The assumption of normality of residuals was verified with the QQ plot with confirmatory results and the analysis of paired multiple comparisons was performed by means of the Bonferroni test.

The results were presented as mean and standard deviation. The differences in which the probability of rejecting the null hypothesis was less than 5% (*p* < 0.05) were considered as statistically significant both intragroup and intergroup at each moment separately.

Intention-to-treat analysis was adopted. For the cases in which treatment was interrupted, the patients were first invited to only perform the assessments. For the patients who refused to return the assessments, the previously collected data were repeated in the subsequent evaluations.

The study complied with the mandatory ethical requirements, respecting the guidelines and regulatory rules for research with human beings.

## Results

Clinical characteristics, demographic information and laboratory characteristics before the start of the trial (baseline) were statistically similar for the study sample and are shown in Table 1. The results suggest the homogeneity between the groups and that randomization was not compromised by discrepancies among the groups.

**Table 1:** Clinical characteristics, demographic information before the start of the trial (baseline) according to the groups

Variable	Calcium group 500 mg/day	Calcium group 1,500 mg/day	Placebo Group	*P*-value
	Mean (SD)	Mean (SD)	Mean (SD)	
Age (years)	30.2 (5.1)	30.4 (5.1)	28.6 (4.7)	0.327
Education (years of study)	12.4 (5.5)	12.4 (4.6)	13.9 (5.3)	0.427
Gestational age (weeks)	26.9 (4.0)	26.3 (4.7)	28.2 (5.0)	0.299
Initial weight (Kg)	94.2 (17.3)	86.5 (16.3)	87.7 (17.9)	0.227
Initial BMI (Kg/m^2^)	35.7 (5.9)	32.6 (7.1)	32.7 (6.2)	0.161
Dietary calcium (g)	531.5 (315.7)	731.5 (477.0)	569.7 (310.1)	0.135
Initial SBP (mmHg)	131.6 (13.8)	131.5 (11.2)	127.8 (12.6)	0.416
Initial DBP (mmHg)	84.2 (9.3)	83.8 (13.2)	81.3 (8.9)	0.531
Triglyceride, mg/dl	224.9 (18.1)	211.2 (15.1)	137.8 (8.9)	0.075
Cholesterol, mg/dl	201.0 (6.9)	235.8 (8.0)	227.1 (6.9)	0.195
LDL-c, mg/dl	101.7 (5.7)	123.8 (6.9)	116.3 (5.8)	0.063
HDL-c, mg/dl	62.8 (3.4)	65.6 (3.5)	76.6 (3.7)	0.393
Hs-CRP, mg/dl	14.3 (1.8)	10.5 (1.3)	3.3 (0.3)	0.268
Ionized calcium, mg/dl	9.1 (0.2)	9.1 (0.2)	9.4 (0.2)	0.832

ANOVA – independent samples / ^#^ Chi-square. *SBP* systolic blood pressure, *DBP* diastolic blood pressure, *BMI* body mass index, *LDL-c* low-density lipoprotein cholesterol, *HDL-c* high-density lipoprotein cholesterol, *hs-CRP* ultrasensitive C-reactive protein

### Signaling of the purinergic ectoenzymes

**Fig. 2:**
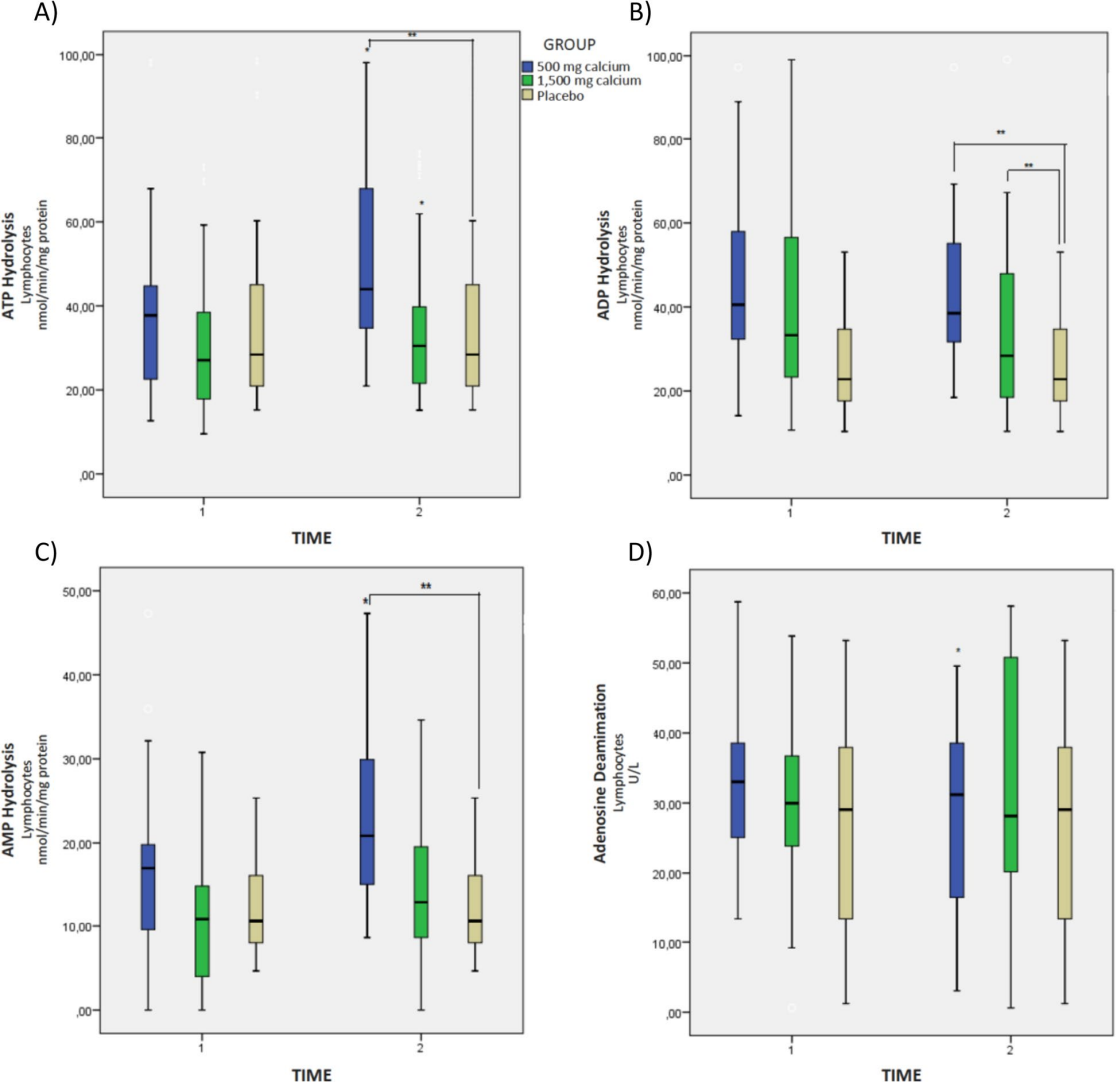
Activity of the purinergic ectoenzymes in lymphocyte samples from hypertensive pregnant women on calcium supplementation. **A** ATP Hydrolysis, **B** ADP Hydrolysis, **C** AMP Hydrolysis, and **D** Ado Deamination. Reference. The laboratory protocol to perform the analysis is described in the methodology. The data were expressed as mean ± standard deviation (GLMM, followed by the Bonferroni post-test, * indicates *p* < 0.05) intragroup, ** indicates *p* < 0.05 intergroup

We investigated the effect of calcium on the pregnant women's purinergic signaling. Figure 1 shows the activity of the purinergic ectoenzymes in lymphocyte samples. The NTPDase activity is observed in relation to substrates ATP, ADP, AMP and ADA and the significant increase of ATP hydrolysis in the group supplemented with 500 mg calcium/day (*p* < 0.05) is verified, when compared to the placebo group (Fig. 2A). There was also a significant increase in this nucleotide in the intragroup analysis among the participants from the lower dose group.

As for the NTPDase activity in relation to the ADP substrate and the ecto-5’-nucleotidase activity, an increase in the hydrolysis of the ADP and AMP nucleotides was observed in the hypertensive pregnant women after supplementation with 500 mg calcium/day and 1,500 mg calcium/day (*p* < 0.05) in relation to the placebo group (Fig. 2B and C). The activity of ADA was significant in the intragroup analysis for the calcium 500 mg/day group after 06 weeks of supplementation (Fig. 2D).

**Fig. 3:**
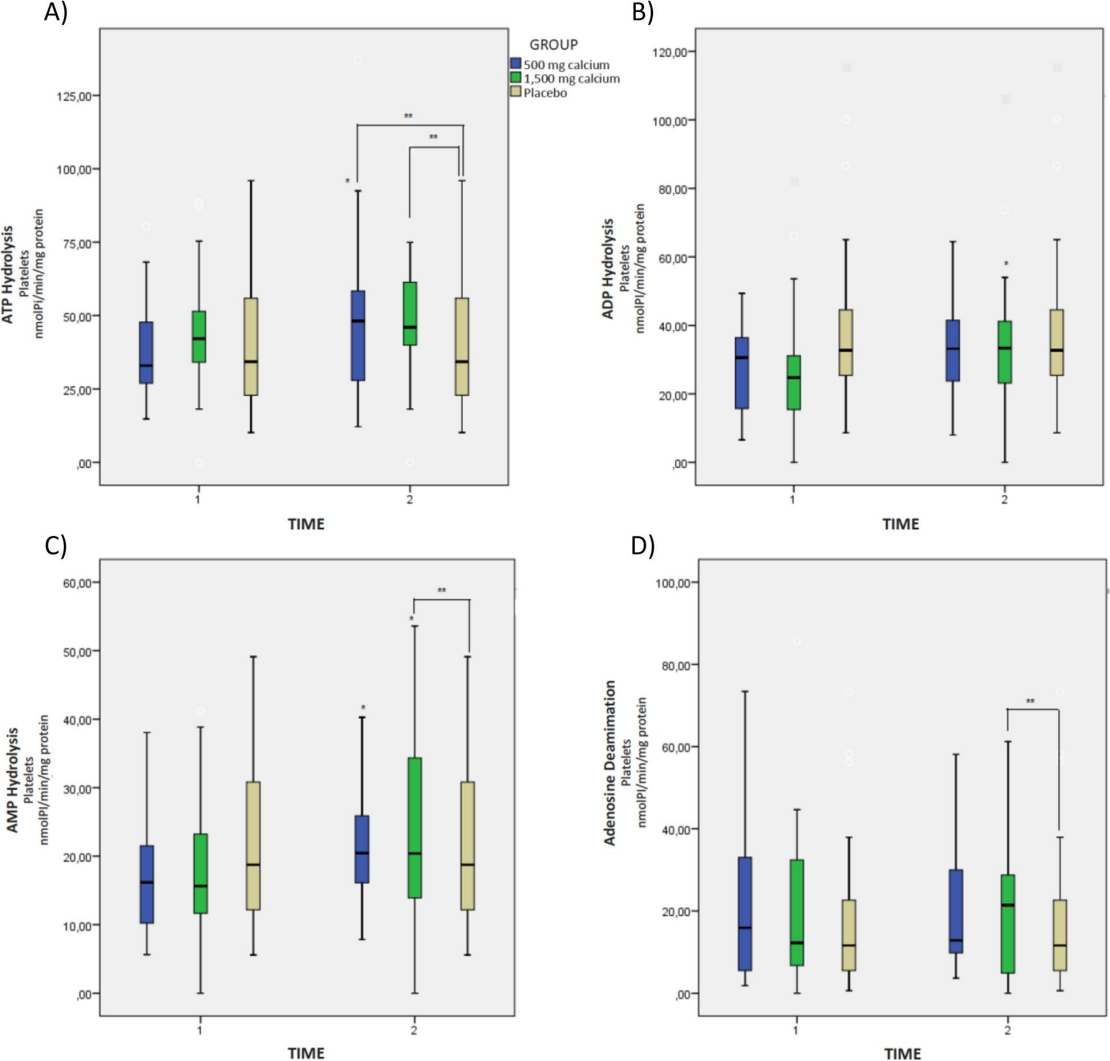
Activity of the purinergic ectoenzymes in platelet samples from hypertensive pregnant women on calcium supplementation. **A** ATP Hydrolysis, **B** ADP Hydrolysis, **C** AMP Hydrolysis, and **D** Ado Deamination. Reference. The laboratory protocol to perform the analysis is described in the methodology. The data were expressed as mean ± standard deviation (GLMM, followed by the Bonferroni post-test, * indicates *p* < 0.05) intragroup, ** indicates *p* < 0.05 intergroup

Figure 3 represents the activity of the purinergic ectoenzymes in platelet samples. We verified increased NTPDase activity with the ATP substrate in the hypertensive pregnant women after 500 mg calcium/day and 1,500 mg calcium/day supplementation when compared to the placebo group (*p* < 0.05) (Fig. 2A). As for the NTPDase activity in relation to the ADP substrate and the ecto-5’-nucleotidase activity, there was an increase in the hydrolysis of ADP and AMP nucleotides for the group supplemented with 1,500 mg calcium/day in the intragroup and intergroup analysis (Fig. 2B and C). The activity of ADA was significant for the calcium 1,500 mg/day (*p* < 0.05) after the intervention, in relation to the placebo group (Fig. 3D).

### Quantification of the interleukins and inflammatory response

**Fig. 4:**
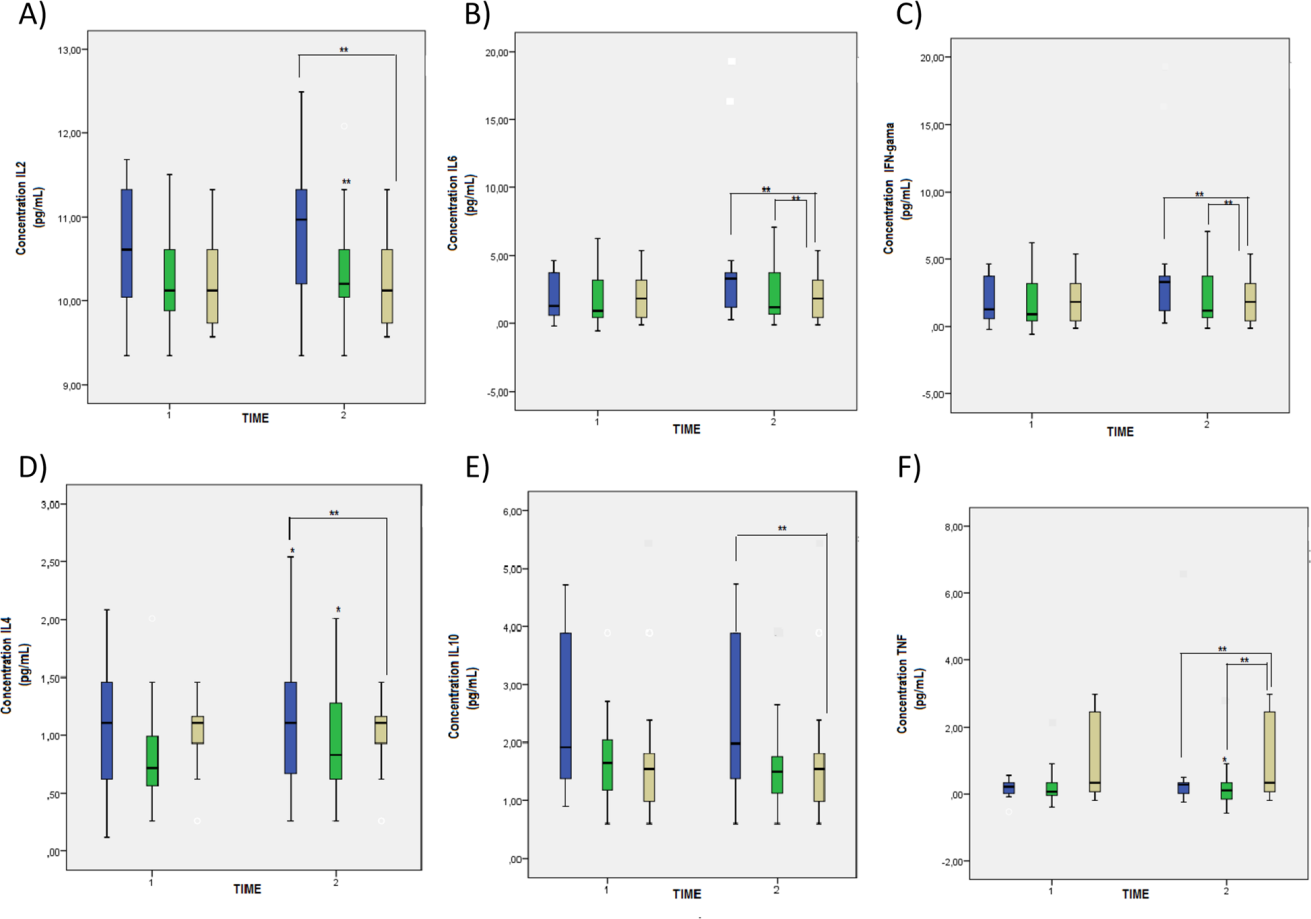
Concentration of pro- and anti-inflammatory interleukins in hypertensive pregnant women on calcium supplementation. **A** IL-2 concentration, **B** IL-6 concentration, **C** Interferon gamma (INF-gamma) concentration, **D** IL-4 concentration, **E** IL-10 concentration, **F** Tumor necrosis factor (TNF) concentration. Reference. The laboratory protocol to perform the analysis is described in the methodology. The data were expressed as mean ± standard deviation (GLMM, followed by the Bonferroni post-test, * indicates *p* < 0.05) intragroup, ** indicates *p* < 0.05 intergroup

The evaluation of the interleukins is represented in Fig. 4, where it can be seen that the pro-inflammatory interleukins, IL-2, IL-6 and interferon-ɣ (4A, 4B and 4C) presented significant differences between the groups (*p* < 0.05). In the intragroup analysis, IL-2, IL-6 and interferon-ɣ were lower after 6 weeks of supplementation in the 1,500 mg calcium/day group when compared to the placebo group. However, these markers presented higher concentrations in the group that received the minimum calcium supplementation.

On the other hand, the anti-inflammatory interleukins presented higher concentrations in the group supplemented with 500 mg calcium/day (*p* < 0.005) (4D, 4E and 4F). After 6 weeks of supplementation it was possible to observe a significant increase in IL-4 in the intragroup analysis (*p* < 0.005) both for the 500 mg calcium/day group and for the 1,500 mg calcium/day group. The IL-10 concentration was higher in the group that received low dosages when compared to the placebo group. The difference in the TNF concentration was significant between the groups, higher in the placebo group.

### Oxidative stress parameters

**Fig. 5:**
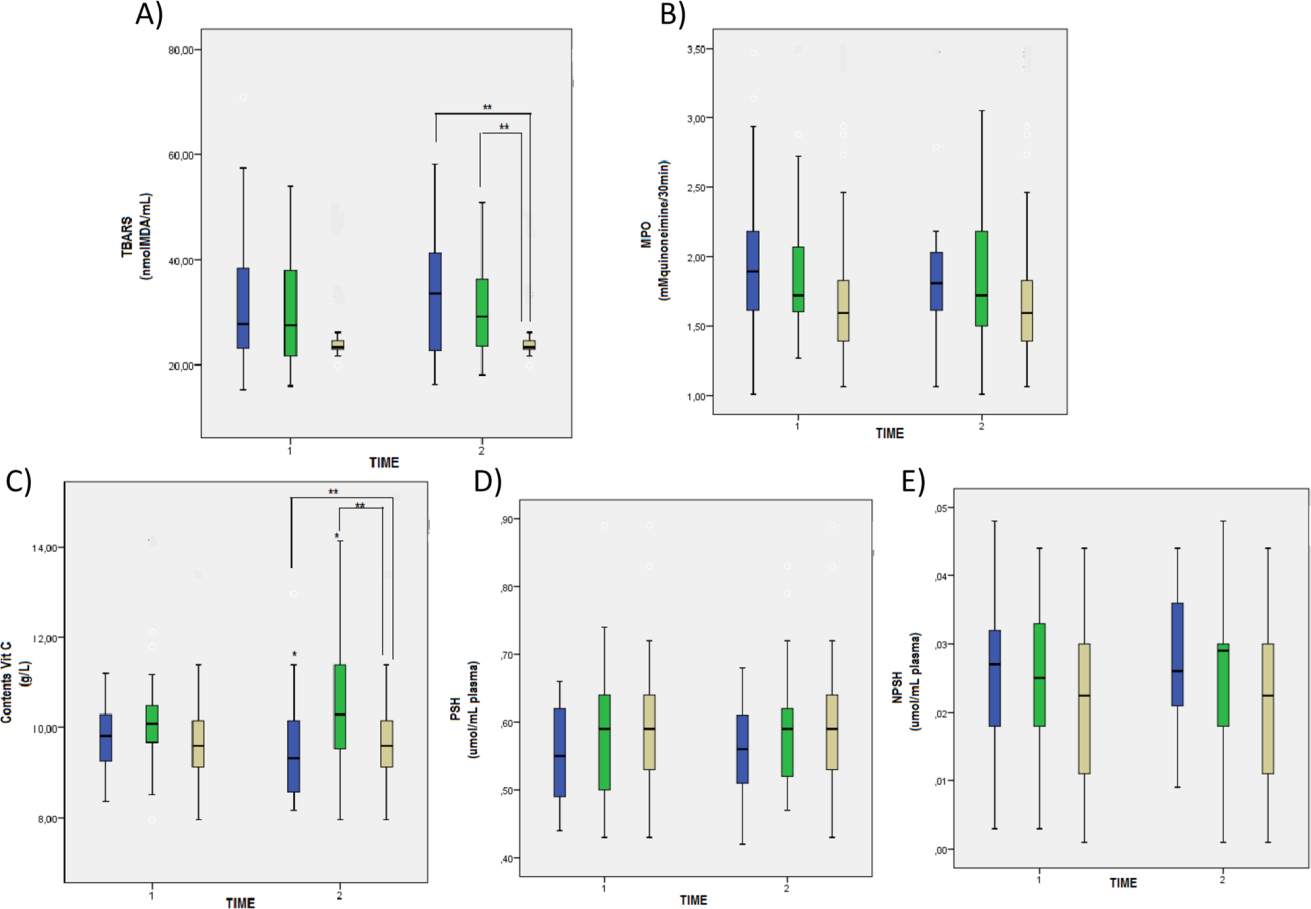
Oxidative profile markers in hypertensive pregnant women on calcium supplementation. **A** Thiobarbituric acid reactive substances (TBARS) concentration, **B** Myeloperoxidase (MPO) concentration, **C** Vitamin C content, **D** Protein thiol group (PSH) concentration, (E) Non-protein thiol group (NPSH) concentration. Reference. The laboratory protocol to perform the analysis is described in the methodology. The data were expressed as mean ± standard deviation (GLMM, followed by the Bonferroni post-test, * indicates *p* < 0.05) intragroup, ** indicates *p* < 0.05 intergroup

The oxidative stress parameters are represented in Fig. 4. There were significant differences (*p* < 0.005) between the groups in the TBARS pro-oxidant marker, with an increase for the 500 mg calcium/day and 1,500 mg calcium/day groups when compared to the placebo group (Fig. 5A). There were no significant differences for the MPO marker (Fig. 5B).

As for the antioxidant markers, the difference was in the Vitamin C content, observing an increase of this marker in the group supplemented with 500 mg calcium/day and 1,500 mg calcium/day when compared to the placebo group (Fig. 5C). In addition, there was a significant increase in the intragroup analysis for the individuals from the calcium 500 mg/day group after 06 weeks of supplementation (*p* < 0.05). No statistical differences were observed for the other antioxidant markers (*p* > 0.05) (Fig. 5D and E).

The secondary outcomes at the end of sex weeks are shown in Table 2. There was no difference between the groups and no worsening of birth conditions.

**Table 2:** Secondary outcomes after 08 weeks of supplementation between the intervention groups

Variable	500 mg/day Group	1.500 mg/day Group	Placebo Group	*P*-value
Birth weight (g)	2.813 (0.69)	2.982 (0.43)	3.233 (0.57)	0.223
Gestational age (weeks)	37.3 (2.2)	37.6 (2.3)	37.9 (1.1)	0.608
Premature delivary	7 (9.0)	6 (7.7)	4 (5.1)	0.237^#^
Neonatal ICU	5 (6.4)	2 (2.6)	3 (3.8)	0.312^#^

ANOVA – independent samples / ^#^ Chi-square

## Discussion

In this study we examined the effects of low and high calcium dosages on the biomarkers of the purinergic system, inflammation and oxidative stress, which are factors contributing to the vascular and endothelial prognosis of pregnant women at high risk of pre-eclampsia. The ischemia-perfusion seen in pre-eclampsia associated with failure of the compensatory mechanisms resulting in placental dysfunction, oxidative and vascular damage can be minimized with the use of calcium. Large randomized and placebo-controlled trials discovered that calcium supplementation prevents the risk of pre-eclampsia [24–26].

Calcium may have stimulated the increase in the activity of the purinergic ectoenzymes in the hypertensive pregnant women participating in our study, and this can have beneficial effects on vasculature, including vasodilation and hypertension control. The purpose of the increase in this enzymatic activity is to preserve vascular permeability. ATP hydrolysis into adenosine ensures anti-inflammatory action in order to avoid pathological effects induced by extracellular ATP [27]. In other words, activation of the inflammatory response indicates the effect of calcium on activation of these cells and on the reduction in the pressure levels. Low calcium doses such as 500 mg/day were already sufficient to promote a satisfactory vasoactive and anti-inflammatory function.

The activity of ADA can be an interesting parameter to assess progression of the disease. Both in lymphocytes and in platelets, the activity of ADA was significant in the different dosages when compared to the placebo group. Other studies also verified that the activity of ADA seems to be effective in reducing the amount of extracellular ATP [27, 28].

Maintenance of adequate activity of the purinergic system allows for ideal concentrations of the nucleotides, which have predefined functions during the pregnancy-puerperal cycle. ATP and ADP promote uterine contraction, by the action of prostaglandins induced by the P2Y-type receptors, also acting on endometrial cell proliferation and differentiation in the postpartum period [29].

In addition, the adenosine released from the placenta acts as a thermogenesis inhibitor before birth, assisting in regulation of fetal metabolism [30]. Extracellular nucleotides also act as regulators of the ion efflux in the placenta, allowing correct perfusion and adequate fetal growth during gestation [31]. On the other hand, placental hypoperfusion is linked to an imbalance in angiogenesis, resulting in vascular endothelial damage, cardiovascular complications and an exaggerated inflammatory response [30, 31].

In the evaluation of the inflammatory markers in our study, we noticed that pro-inflammatory interleukins IL-2 and IL-6 presented lower concentrations in the group that received high calcium doses. The effect of calcium in high dosages is well-known for reducing the inflammatory damages in hypertensive pregnant women [26–28].

IL-2 and IL-6 are inflammatory markers found during pregnancy due to the implantation, invasion and vascularization of trophoblast cells in the maternal endometrium. In fact, the presence of these markers is essential both in maintenance of the pregnancy and in progression of the delivery [1]. However, the balance between the pro- and anti-inflammatory conditions needs to be controlled by means of several regulating mechanisms, and calcium may be yet another of these satisfactory mechanisms [32].

According to these findings, the concentrations of anti-inflammatory cytokines such as IL-4, IL-10 and TNF were higher in the supplemented groups when compared to the placebo group, reinforcing the effect of calcium in reducing both endothelial dysfunctions and inflammatory processes in at-risk pregnant women. The anti-inflammatory activity of calcium is presumed to cause remodeling via the activated immune responses, leading to an improvement in endothelial dysfunction [33].

It is possible that the pre-existing altered vascular function arising from an inadequately perfused placenta is one of the fundamental causes of the inflammation and oxidative stress response. Among the oxidative stress markers there were significant differences between the supplemented and placebo groups, with an increase in the concentration of the TBARS oxidant in the groups that were supplemented with calcium, both at the 500 mg/day and at the 1,500 mg/day dosage. On the other hand, the antioxidant analyzed, Vitamin C, presented higher concentrations in the groups that received calcium, regardless of the dosage [34, 35].

It is possible that the increase in oxidative stress may have been counterbalanced by the increase in the synthesis of antioxidants. In recent years, oxidative stress biomarkers were detected in the blood from women with pre-eclampsia (3). Other studies have also identified increased levels of thiobarbituric acid reactive substances (TBARS), measured as production of malondialdehyde (MDA), a degradation product of lipid peroxidation, in erythrocytes, blood plasma and placental tissue [34, 35].

However, daily calcium supplementation appears to favor increased Vitamin C synthesis with a possible reduction in vascular damage, given the similarity between the groups in this study before supplementation. It can be predicted that the satisfactory effect of the increased antioxidant synthesis was the result of continuous calcium use due to its antioxidant and anti-inflammatory properties [36].

Despite a more obvious reduction for vascular and inflammatory damage in the group that received higher calcium doses (1,500 mg/day), the effects of calcium were also satisfactory in the group that was administered lower dosages. Lower dosages can result in fewer side effects, greater adherence to the treatment, easier intake and lower costs for the health services, which should be especially considered in still developing countries [26–28].

After supplementation, there was no association between the received dosages and low birth weight and/or prematurity. Regardless of the received dosage, the disease in question did not worsen the exposure to risk or worsen neonatal outcomes.

Thus, the need for calcium supplementation in the population of hypertensive pregnant women is reinforced, in order to reduce the potential risk of complications from cardiovascular diseases.

## Conclusions

Considering the probability of low adherence among the women with high supplementation doses, at least the minimum effective dose should be considered and guaranteed. It is important to emphasize that calcium supplementation in any dosage is not part of the health services' protocols in most Brazilian regions and states, just as it is not where this study was conducted, which can be another argument in favor of encouraging prescription of the supplement in the clinical practice.

After 6 weeks of supplementation, there was no association between the supplemented dosages and low birth weight, prematurity or worsening of the birth outcomes. Regardless of the dosage received, the disease in question did not worsen risk exposure or the neonatal outcomes. Thus, the need for calcium supplementation in the population of hypertensive pregnant women is reinforced, in order to reduce the potential risk of complications due to vascular diseases.

The low prevalence of severe complications was expected because two groups were supplemented with calcium, which is known to reduce severe complications in hypertensive disorders. Given the similarity of the groups before the clinical decision, the favorable outcomes found can be the result of the supplemented calcium. Daily calcium supplementation after 6 weeks significantly increased the activity of the purinergic system ectoenzymes, the concentration of anti-inflammatory interleukins and the concentration of antioxidant markers, contributing to the reduction of vascular damage and inflammatory processes caused by the systemic reaction of gestational hypertension.

There was an improvement in the outcomes studied for the group that received higher calcium doses (1,500 mg/day), but the effects of calcium in a lower dosage (500 mg/day) were also satisfactory in reducing vascular damage. Our study evidences the need for prevention and control of the inflammatory profile and antioxidant status in the clinical practice for at-risk pregnant women. Therapies to alleviate symptoms and maintain pregnancy are necessary to prolong gestation.

### Supplementary Information

**Additional file 1. **Supplementary Material. **Figure 01.** Flow diagram of the study design and participant allocation. **Table 1.** Clinical characteristics, demographic information before the start of the trial (baseline) according to the groups.

## Data Availability

All data generated or analysed during this study are included in this published article.
