# Supplementary material for: Effects of calcium supplementation on changes in the IL2, IL4, IL6, IL10 axes and oxidative stress in pregnant women at risk for pre-eclampsia
Source: BMC Pregnancy Childbirth. 2024 Jan 20;24:71. doi: 10.1186/s12884-023-06235-8 (PMC10799391; doi:10.1186/s12884-023-06235-8)
Supplement: Supplementary file 1 — Additional file 1. Supplementary Material. Figure 01. Flow diagram of the study design and participant allocation. Table 1. Clinical characteristics, demographic information before the start of the trial (baseline) according to the groups. [file 12884_2023_6235_MOESM1_ESM.docx]

Supplementary Material.

Results

In the recruitment and allocation of the 193 eligible participants, 05 were excluded before randomization for not meeting the inclusion criteria, 11 refused to participate and 02 had severe anemia. Accordingly, 175 pregnant women were randomized and randomly allocated into 3 groups, preliminarily divided into placebo group (n=59), calcium group 500mg/day (n=58) and calcium group 1,500mg/day (n=58).

At the follow-up after 6 weeks, there were 44 losses, 12 in the placebo group, 15 in the calcium group 500mg/day and 17 in calcium group 1,500mg/day. In the analysis of the total number of pregnant women who started the research protocol, 83 completed all stages (Figure 01). Supplementary Material displays the flow chart of study participants according to CONSORT 2010. For patients who did not attend reassessments, data from the last assessment were repeated to perform the intention-to-treat analysis.

Clinical characteristics, demographic information and laboratory characteristics before the start of the trial (baseline) were statistically similar for the study sample and are shown in Table 1. The results suggest the homogeneity between the groups and that randomization was not compromised by discrepancies among the groups. At the end of the study, after 8 weeks, low treatment adherence and loss with regard to follow-up were significant for the group with maximal supplementation.

Assessed for eligibility (n= 193)

Randomized (n= 175)

Excluded (n=18)

Not meeting inclusion criteria (n=05)

Declined to participate (n=11)

Other reasons (n=02)

Placebo Group

(n=59 received placebo)

Maximum Intervention Group (n=58 received calcium 1500mg/day)

Minimum Intervention Group (n=58 received calcium 500mg/day)

Analysed (n=32)

Discontinued intervention (n=12)

- placental insufficiency (n=02)

- polyhydramnios (n=02)

- premature rupture of membranes (n=01)

- severe preeclampsia (n=01)

- premature placental abruption (n=06)

Discontinued intervention (n=15)

- placental insufficiency (n=07)

- polyhydramnios (n=03)

- premature rupture of membranes (n=02)

- premature placental abruption (n=03)

Discontinued intervention (n=17)

- placental insufficiency (n=04)

- polyhydramnios (n=04)

- severe preeclampsia (n=03)

- premature placental abruption (n=06)

Discontinued intervention (n=15)

- placental insufficiency (n=03)

- polyhydramnios (n=04)

- premature rupture of membranes (n=05)

- premature placental abruption (n=03)

Discontinued intervention (n=16)

- placental insufficiency (n=02)

- polyhydramnios (n=03)

- severe preeclampsia (n=02)

- premature placental abruption (n=09)

Analysed (n=27)

Discontinued intervention (n=17)

- placental insufficiency (n=04)

- premature rupture of membranes (n=07)

- severe preeclampsia (n=03)

- premature placental abruption (n=03)

Analysed (n=24)

**Figure 01 -** Flow diagram of the study design and participant allocation.

**Table 1** Clinical characteristics, demographic information before the start of the trial (baseline) according to the groups.

| Variable | Calcium group 500mg/day  (n=58) | Calcium group  1,500mg/day  (n=58) | Placebo  Group  (n=59) | p-value |
| --- | --- | --- | --- | --- |
|  | Mean (SD) | Mean (SD) | Mean (SD) |  |
| Age (years) | 30.2 (5.1) | 30.4 (5.1) | 28.6 (4.7) | 0.327 |
| Education (years of study) | 12.4 (5.5) | 12.4 (4.6) | 13.9 (5.3) | 0.427 |
| Gestational age (weeks) | 26.9 (4.0) | 26.3 (4.7) | 28.2 (5.0) | 0.299 |
| Initial weight (Kg) | 94.2 (17.3) | 86.5 (16.3) | 87.7 (17.9) | 0.227 |
| Initial BMI (Kg/m^2^) | 35.7 (5.9) | 32.6 (7.1) | 32.7 (6.2) | 0.161 |
| Dietary calcium (g) | 531.5 (315.7) | 731.5 (477.0) | 569.7 (310.1) | 0.135 |
| Initial SBP (mmHg) | 131.6 (13.8) | 131.5 (11.2) | 127.8 (12.6) | 0.416 |
| Initial DBP (mmHg) | 84.2 (9.3) | 83.8 (13.2) | 81.3 (8.9) | 0.531 |
| Triglyceride, mg/dl | 224.9 (18.1) | 211.2 (15.1) | 137.8 (8.9) | 0.075 |
| Cholesterol, mg/dl | 201.0 (6.9) | 235.8 (8.0) | 227.1 (6.9) | 0.195 |
| LDL-c, mg/dl | 101.7 (5.7) | 123.8 (6.9) | 116.3 (5.8) | 0.063 |
| HDL-c, mg/dl | 62.8 (3.4) | 65.6 (3.5) | 76.6 (3.7) | 0.393 |
| Hs-CRP, mg/dl | 14.3 (1.8) | 10.5 (1.3) | 3.3 (0.3) | 0.268 |
| Ionized calcium, mg/dl | 9.1 (0.2) | 9.1 (0.2) | 9.4(0.2) | 0.832 |
|  |  |  |  |  |
| After 8 weeks | n (%) | n (%) | n (%) |  |
| Low treatment adherence | 4 (2.2) | 14 (8)^#^ | 3 (1.7) | **0.026^#^** |
| Follow-up loss | 31 (17.7) | 34 (19.4) | 27 (15.4) | 0.890 |

ANOVA – independent samples / ^#^ Chi-square. SBP, systolic blood pressure; DBP, diastolic blood pressure; BMI, body mass index; LDL-c, low-density lipoprotein cholesterol; HDL-c, high-density lipoprotein cholesterol; hs-CRP, ultrasensitive C-reactive protein.
